# Numerical investigation of depth profiling capabilities of helium and neon ions in ion microscopy

**DOI:** 10.3762/bjnano.7.168

**Published:** 2016-11-17

**Authors:** Patrick Philipp, Lukasz Rzeznik, Tom Wirtz

**Affiliations:** 1 Advanced Instrumentation for Ion Nano-Analytics (AINA), MRT Department, Luxembourg Institute of Science and Technology (LIST), 41 rue du Brill, L-4422 Belvaux, Luxembourg

**Keywords:** atomic mixing, depth profiling, helium ion microscopy, ion bombardment, numerical simulations, polymers, SDTRIMSP

## Abstract

The analysis of polymers by secondary ion mass spectrometry (SIMS) has been a topic of interest for many years. In recent years, the primary ion species evolved from heavy monatomic ions to cluster and massive cluster primary ions in order to preserve a maximum of organic information. The progress in less-damaging sputtering goes along with a loss in lateral resolution for 2D and 3D imaging. By contrast the development of a mass spectrometer as an add-on tool for the helium ion microscope (HIM), which uses finely focussed He^+^ or Ne^+^ beams, allows for the analysis of secondary ions and small secondary cluster ions with unprecedented lateral resolution. Irradiation induced damage and depth profiling capabilities obtained with these light rare gas species have been far less investigated than ion species used classically in SIMS. In this paper we simulated the sputtering of multi-layered polymer samples using the BCA (binary collision approximation) code SD_TRIM_SP to study preferential sputtering and atomic mixing in such samples up to a fluence of 10^18^ ions/cm^2^. Results show that helium primary ions are completely inappropriate for depth profiling applications with this kind of sample materials while results for neon are similar to argon. The latter is commonly used as primary ion species in SIMS. For the two heavier species, layers separated by 10 nm can be distinguished for impact energies of a few keV. These results are encouraging for 3D imaging applications where lateral and depth information are of importance.

## Introduction

Ion bombardment of polymer samples has been studied for various applications related to surface modifications and surface analysis. Ion bombardment of polymers allows to change the electric conductivity of polymers [1]. A reduction of the band gap along with increasing photo- and electrical conductivity is observed for C^+^ implantation into poly(methyl methacrylate) (PMMA), which is related to the formation of carbon clusters with a polyaromatic structure [2]. Potential applications include transistor-like switches, optical switches, and photodiodes. Cu implanted polycarbonate (PC) has a potential application in strain-sensing technologies and electrical biosensor [3]. Modification of the mechanical properties of the polymer surface has also been studied. N^+^ implantation in PC leads to a harder surface by disordering the surface structure and forming hydrogenated amorphous carbon [4]. Similarly, Ga^+^ irradiation of polydimethylsiloxane (PDMS) results in micro- and nanopatterns with controlled stiffness for potential applications in tissue engineering [5]. Overall, the properties depend on the initial polymer structure and the implanted species. For C^4+^ and O^4+^ irradiation of poly(etheretherketone) (PEEK), polypropylene (PP), polyethyleneterephthalate (PET), and PC, PP has the lowest resistance to irradiation damage while the most important compositional changes occur in PC [6]. For sensors, B^+^ irradiation of polyimide (PI) and polyethersulfone improves the moisture uptake in the films [7]. Further applications include metal adhesion on polymer surface [8], novel inorganic films by ion bombardment of polymers [9], surface morphology for biocompatibility [10], etch resistance of polymers [11], processes for graphene based electronics, the preparation of ultra-hydrophobic fluorine polymers by Ar-ion bombardment [12], and ion implantation to form nanoparticles inside polymers [13].

Common to the different applications is the damage created by the energetic ions upon their implantation in the polymer sample. The ion beam induces chemical and structural modifications in the polymers, chain scission and bond breaking as well as crosslinking depending on the chemical composition and the structure of the polymer chain [7]. In general, heterocyclic groups are more resistant to electron excitation, opening the possibility of transformations in the chain. The outcome might be also different depending on whether electronic or nuclear stopping is prevalent. The latter depends on the choice of the primary ion species and the impact energy [7]. The release of small molecules is the consequence of the damage formation. The emission of H_2_, CH_4_, C_2_H_2_, C_3_H_5_, etc. has been observed for 100 keV He^+^ and 200 keV Ar^+^ bombardment of polyethylene (PE) and polystyrene (PS) [14]. Similarly, H_2_, C_2_H_2_, CO and CO_2_ are typically emitted from implanted PI [15]. The degassing leads to an increase of the carbon concentration in the polymers, hence a carbonisation of the polymer is observed [16], resulting in a buried carbon-rich layer in polymers [17]. This is of significant interest because it goes along with large changes in structure and properties of the polymers. In this context, the formation of carbon clusters has been observed upon 100 keV B^+^ and N^+^ implantation of PE and polyamide (PA), but is not limited to these polymers [16]. The formation of saturated hydrocarbons is observed when bombarding PP and polybutylene with ions [18]. At first degassing of small molecules goes along with the transformation of functional groups and cross-linking and the formation of “pre-carbon” structures. Next, the nucleation and growth of carbon-enriched structures is observed, followed by the aggregation of carbon clusters up to formation of quasi-continuous carbon-rich buried layer forming a network of conjugated bonds. Finally, a transition of a carbonised phase to an amorphous carbon or graphite-like material occurs [13]. Another interesting field of investigation is the formation of nanoparticles by ion implantation. By adapting the impact energy, the implantation depth can be well controlled, and so the localisation of the nanoparticles below the surface. Their formation is observed for concentrations of the implanted species beyond the solubility limit. For most polymers, nucleation starts at 10^16^ ions/cm^2^ and at very high fluences a worm-like structure can start to form. However, not all implanted metal atoms end up in the nanoparticles [13]. The nanoparticles are formed due to high cohesive energy of metal atoms and their weak interaction with the polymer chains. The conductivity of the implanted sample increases with increasing fluence. A colour change is observed after implantation due to the formation of nano-sized carbon-rich clusters, along with the enhancement of photoluminescence. Paramagnetic properties can also appear due to changes in the polymer structure. Another method to tune the magnetic properties is the formation of nanoparticles by ion implantation [13].

For ion microscopy, or more specifically secondary ion mass spectrometry (SIMS), analysis of organic samples by sputtering is also one important field of applications and the kind of damage mentioned for previous applications remains the same. However, for depth profiling applications of polymers [19] and biological samples [20], minimising the fragmentation of the polymer chains to retain a maximum of organic information by using cluster primary ions is one field of investigation. Nevertheless, for high lateral resolution, the use of monatomic primary ion species is however required. For the latter, the detection of small secondary ion clusters is only possible due to the large fragmentation of the organic molecules. In these studies, the build-up of electric charges under ion bombardment, its correlation with the changes in surface composition of polymers and the dielectric break-down as a function of fluence has been investigated [19–20]. In the same context, the sputtering and collection of polymer fragments on a metal substrate and their subsequent analysis by SIMS can be mentioned [21–22].

In this work we investigate how light rare gas ion irradiation affects damage formation and sputter processes in multi-layered polymer samples as a function of ion bombardment conditions and sample composition to evaluate the different conditions for 3D depth profiling capabilities in ion microscopy. Of particular interest is the preferential sputtering and atomic mixing for the different irradiation conditions to determine the depth resolving power for experimental conditions on the helium ion microscope (HIM). As such it contributes to the development of SIMS on the helium ion microscope [23–25] in order to extend its application to organic samples. The interest of this work is not limited to polymer samples, as they are also used as model samples for biological applications. The research work was carried out using the SD_TRIM_SP code [26] which can model the modification of the sample induced by the ion bombardment as a function of fluence and has the ability to model the diffusion of rare gas species in the polymer sample. SRIM, which is based on the same concept, is not suited for this study because the preferential sputtering and atomic mixing are not taken into account, leading to significant discrepancy between experimental and simulation results [13]. Yet, diffusion processes in general are also not taken into account in the SD_TRIM_SP code.

## Results and Discussion

### Initial samples composition

Multilayered samples have been constructed to study the influence of layer composition and structure on the outcome of depth profiling experiments for typical experimental conditions on the HIM. By changing the chemical species present in the different layers, the influence of atomic mass on atomic mixing and the sputtering processes can be studied for different primary ion species. For this reason, layers of polytetrafluoroethylene (PTFE), PS or PMMA have been embedded into PE layers. Compared to molecular dynamics (MD) simulations, possible changes in the sample upon irradiation due to chemical reactions, as they are included in reactive force fields [27–29], are not contained in the model, but SD_TRIM_SP has the advantage to allow for the simulation of high fluences up in the 10^18^ ions/cm^2^ range, i.e., ion fluences used in experiments.

**Figure 1:**
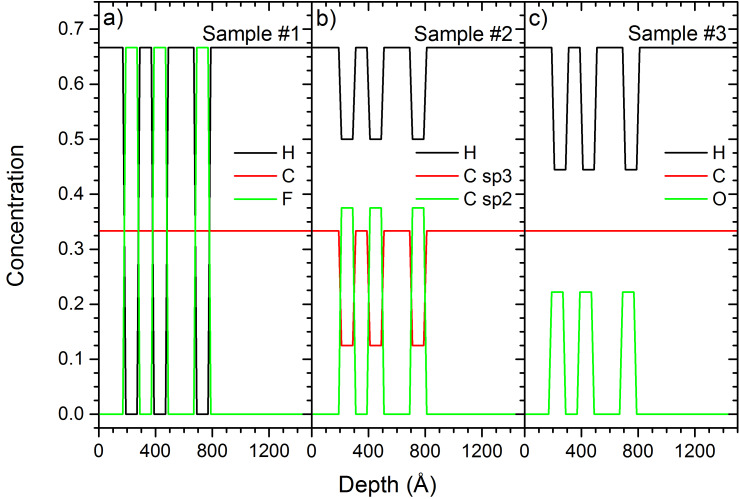
Initial sample composition for a) sample #1, b) sample #2, and c) sample #3. All samples have inter-layer distances of 10 nm and 20 nm.

A detailed description of the different samples is given in Table 1. Figure 1 shows the composition and structure for samples #1 to #3. The three samples have 10 nm thick PTFE, PS or PMMA layers separated by 10 nm or 20 nm PE layers. The bulk material is also PE. For samples #4 to #6 the thickness of the PTFE, PS and PMMA layers and the distance between these layers is changed. For samples #1 and #3 the carbon concentration is constant at 33%. For sample #1 the hydrogen and fluorine concentrations are alternating between 0% and 67% depending on the polymer layer. For sample #3 hydrogen and oxygen concentrations are changing depending on the layer. For sample #2, the concentrations of all species are changing with the layers.

**Table 1:** Layer thicknesses and composition for the different samples irradiated with He^+^, Ne^+^ and Ar^+^ ions.

Layers		Sample #					
		1	2	3	4	5	6

1	Polymer	PE	PE	PE	PE	PE	PE
	Thickness (nm)	20	20	20	20	20	20

2	Polymer	PTFE	PS	PMMA	PTFE	PS	PMMA
	Thickness (nm)	10	10	10	10	10	10

3	Polymer	PE	PE	PE	PE	PE	PE
	Thickness (nm)	10	10	10	20	20	20

4	Polymer	PTFE	PS	PMMA	PTFE	PS	PMMA
	Thickness (nm)	10	10	10	10	10	10

5	Polymer	PE	PE	PE	PE	PE	PE
	Thickness (nm)	20	20	20	40	40	40

6	Polymer	PTFE	PS	PMMA	PTFE	PS	PMMA
	Thickness (nm)	10	10	10	20	20	20
Bulk	Polymer	PE	PE	PE	PE	PE	PE

### Depth profiling capabilities for He^+^ bombardment

**Figure 2:**
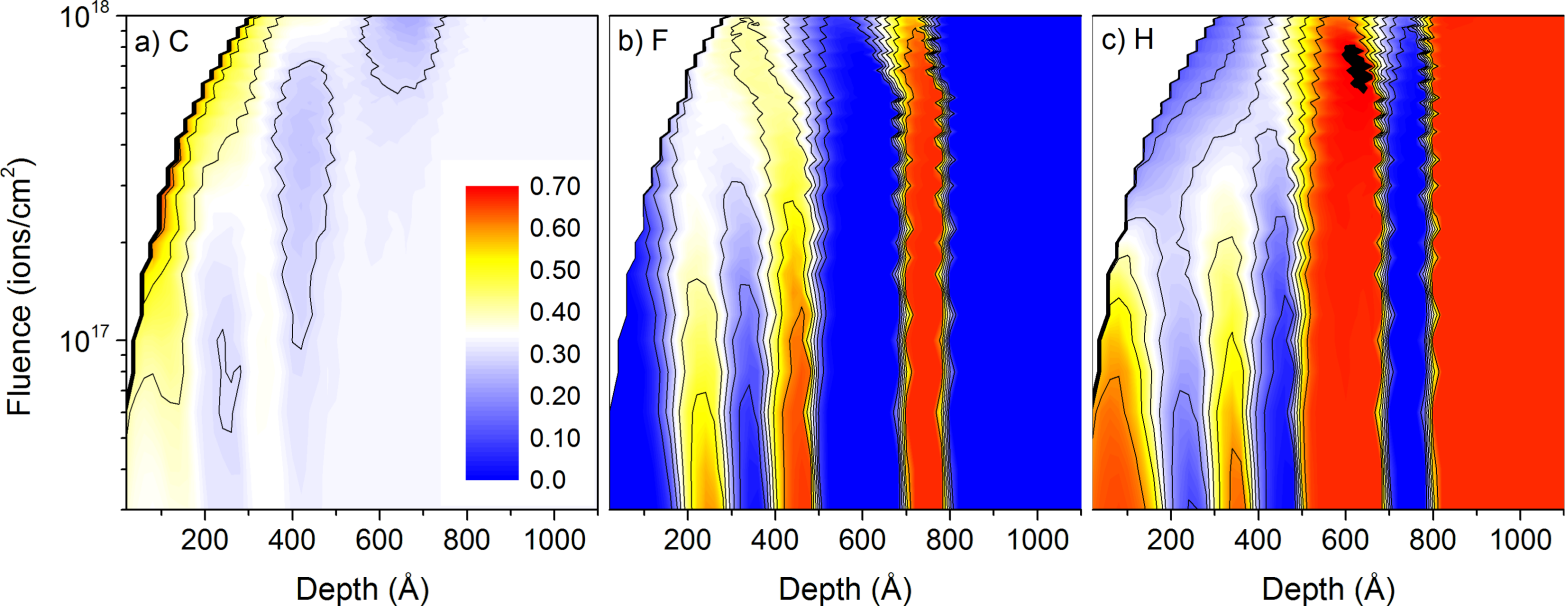
Sample composition as a function of depth and primary ion fluence for 1 keV He^+^ irradiation of sample #1 with the thin-layer configuration: a) for carbon, b) for fluorine, and c) for hydrogen. The colour scale shows concentrations from 0 to 70% and is identical for the three graphs.

Sample numbers given in this section refer to the details on samples given in Table 1. Compared to the other primary ion species used in this work, He^+^ ions have a much smaller mass, leading to much higher implantation depths, significantly smaller sputtering yields and a different atomic mixing. Figure 1 shows the initial concentration profile of carbon, fluorine and hydrogen for sample #1 with alternating PE and PTFE layers. The carbon concentration is constant throughout the whole sample. This changes after 1 keV He^+^ irradiation up to a fluence of 10^18^ ions/cm^2^. The fluorine and hydrogen concentration profiles get gradually blurred out (Figure 2). The large implantation depth combined with the low sputtering yield under He^+^ irradiation produce an important atomic mixing, smearing out the concentration gradients before the different PE and PTFE layers have been sputtered. The fluorine of the first layer has been completely mixed with the surrounding PE layers up to a fluence of 1.5 × 10^17^ ions/cm^2^, and the second layer for a fluence of 3.0 × 10^17^ ions/cm^2^. This shows that the atomic mixing is very effective over a sample depth of about 40 nm. At the same time, the He^+^ implantation leads to some relocation of carbon atoms in the PTFE layer. The initially constant carbon concentration throughout the whole sample gets lowered in these layers and the hydrogen concentration increased. In the PE layers, only hydrogen gets displaced by the atomic mixing because of the large difference in mass between hydrogen and carbon. In the PTFE layers, fluorine and carbon have a similar mass, leading to the atomic mixing of both species. Accumulation of helium inside the sample is avoided by the relatively high diffusion coefficients of helium in polymers [30]. The important atomic mixing combined with the low sputtering yields makes 1 keV He^+^ bombardment unsuited for the depth profiling of 10 nm polymer films.

**Figure 3:**
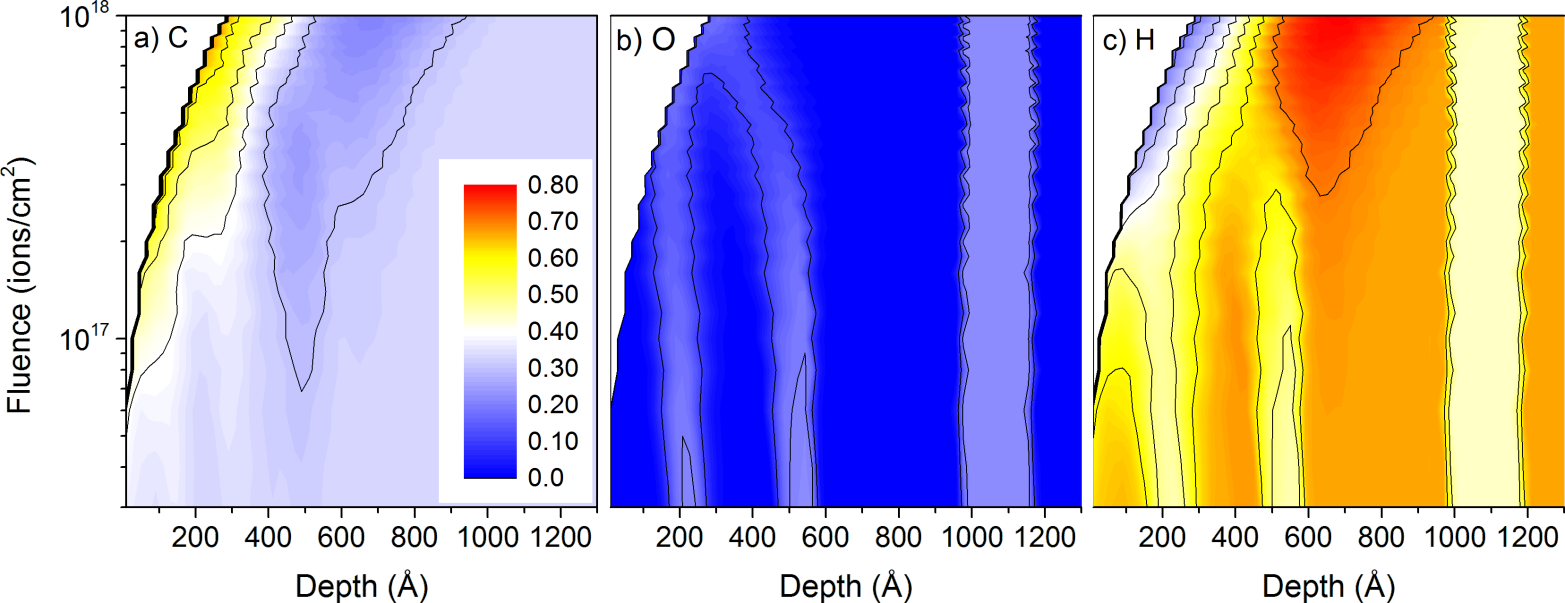
Sample composition as a function of depth and primary ion fluence for 1 keV He^+^ irradiation of sample #6 with the thick-layer configuration: a) for carbon, b) for oxygen, and c) for hydrogen. The colour scale shows concentrations from 0 to 80% and is identical for all species.

The same observation is true for the other multi-layered samples under 1 keV He^+^ irradiation. Changing the composition of the layers does not change the sputtering and mixing mechanisms significantly (Figure 3). For PMMA layers in sample #6, the oxygen concentration profiles get blurred out similarly to the PTFE layers in sample #1. Differences in mixing are related to the larger distance between the first two PMMA layers compared to sample #1 (20 nm for PMMA vs 10 nm for PTFE). Figure 3 shows also clearly that the distance between the different layers is still too small to be differentiated by the 1 keV He^+^ bombardment. The oxygen concentrations at the sample surface are still high when the sputtering of the sample reveals the second PMMA layer. For 20 keV He^+^ irradiation, no data is shown because the mixing is much worse than for 1 keV He^+^ and these conditions are even worse for depth profiling applications of multi-layered polymer samples, or organic samples in general.

### Depth profiling using 20 keV Ne^+^ and Ar^+^

**Figure 4:**
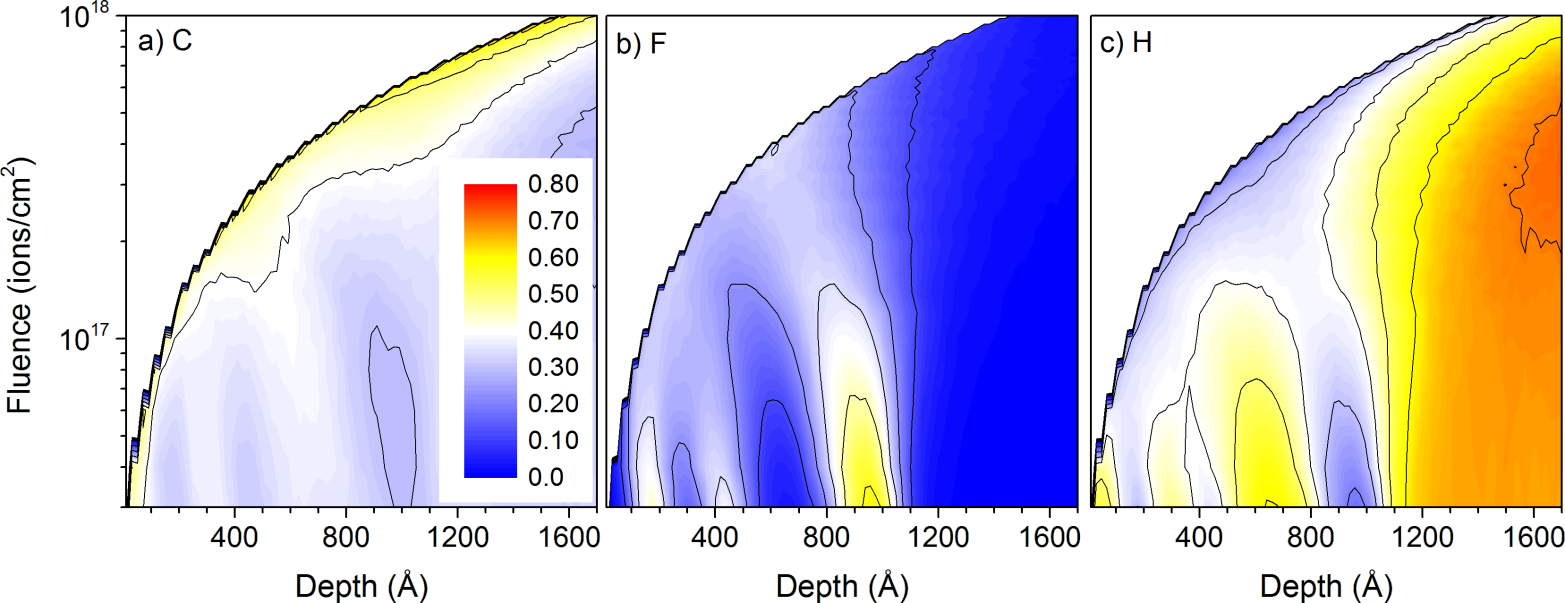
Sample composition as a function of depth and primary ion fluence for 20 keV Ne^+^ irradiation of sample #4 with the thick-layer configuration: a) for carbon, b) for fluorine, and c) hydrogen. The colour scale shows concentrations from 0 to 80% and is identical for all species.

**Figure 5:**
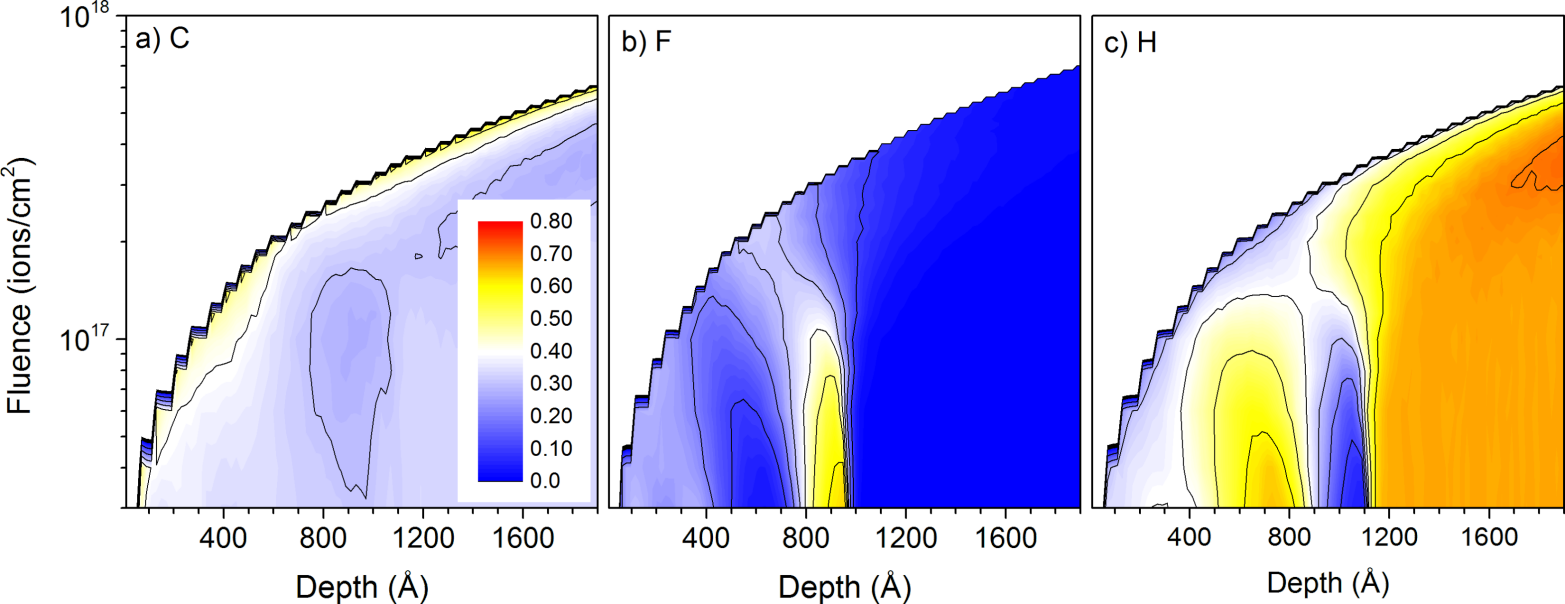
Sample composition as a function of depth and primary ion fluence for 20 keV Ar^+^ irradiation of sample #4 with the thick-layer configuration: a) for carbon, b) for fluorine, and c) hydrogen. The colour scale shows concentrations from 0 to 80% and is identical for all species.

Ne^+^ and Ar^+^ primary ions are much heavier than He^+^ ions, leading to higher sputtering rates and lower implantation depths than the latter. This improves their abilities for depth profiling capabilities. Ar^+^ ions have already been used for applications in SIMS [31–32], especially before the development of cluster ion beams [33]. In this paragraph we are going to compare them to Ne^+^ ions which are going to be used on the HIM for similar applications. Although the difference in mass between neon and argon is significant it is much smaller than between helium and neon or argon, and the modifications in the polymer samples for both primary ion species are comparable. For 20 keV Ne^+^ and Ar^+^ bombardment, polymers sputter 10–100 times faster than for He^+^ ions, leading to the removal of the PTFE layers in sample #4 with PE and PTFE layers for fluences below 8 × 10^17^ ions/cm^2^ (Figure 4 and Figure 5). For both situations, the fluorine surface concentration remains high in between the different PTFE layers, making it impossible to distinguish the layers separated by a 20 nm PE layer in the depth profile. Only for Ar^+^ the fluorine concentration drops in between the PTFE layers separated by 40 nm (Figure 5). Hence, the heavier primary ion is best suited for depth profiling, but the improvement compared to 20 keV Ne^+^ irradiation is not that large.

For 20 keV Ar^+^ ion bombardment, the lower implantation depth reduces the atomic mixing but the surface concentrations of carbon, fluorine and hydrogen are similar to the Ne^+^ irradiation, with the carbon surface increasing to about 60% to 70% and the hydrogen surface concentration getting reduced to about 30% to 40%. The fluorine surface concentration is at maximum around 30% although its initial concentration in the PTFE films is of 67%. So carbon is the only species which gets enriched in the surface region. At maximum implantation depth of the primary ion species, the samples get slightly enriched in hydrogen. It seems it gets pushed by the atomic mixing into this region. Its concentration of about 77% is about 10% above the bulk hydrogen concentration in PE. Similar to He^+^ irradiation, the relatively high diffusion coefficients of neon and argon in the four polymers used in this study avoid the accumulation of rare gases in the different samples.

Similar figures for the other polymer samples are not shown because the outcome is identical to the PE/PTFE layers. The composition and structure of the polymers do not have a significant impact on the sputtering and atomic mixing processes. For the samples with the thin layers, the separation of the layers is even worse.

**Figure 6:**
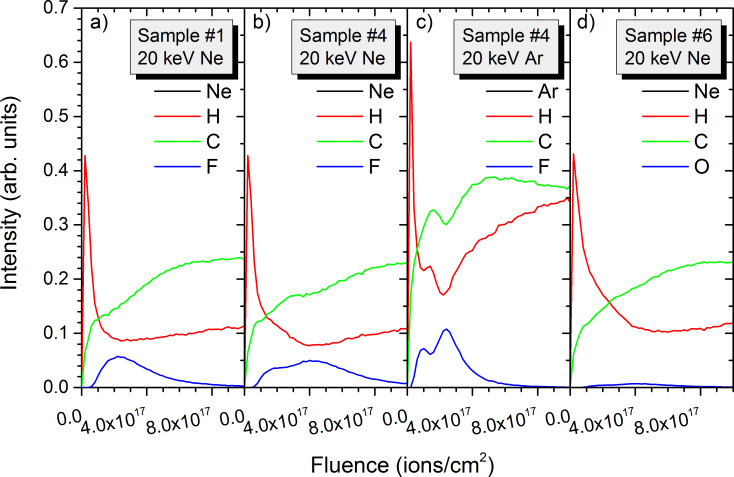
Simulated depth profiles for a) 20 keV Ne^+^ irradiation of sample #1 with 10 nm and 20 nm inter-layer spacing, b) 20 keV Ne^+^ irradiation of sample #4 with 20 nm and 40 nm inter-layer spacing, c) 20 keV Ar^+^ irradiation of sample #4 with 20 nm and 40 nm inter-layer spacing, and d) 20 keV Ne^+^ irradiation of sample #6 with 20 nm and 40 nm inter-layer spacing.

Data from Figure 4 and Figure 5 along with results from samples #1 and #6 is used to reconstruct the depth profiles for the different species present in the polymer samples (Figure 6). Depth profiles are shown for sample #1 with 10 to 20 nm thick layers and sample #3 with 10 to 40 nm thick layers. For sample #1, the fluorine intensity increases at the beginning of the depth profile, reaching its maximum at a fluence of 2.5 × 10^17^ ions/cm^2^ for Ne^+^ irradiation. This corresponds to the end position of the second PTFE layer. The important atomic mixing leads only to a slow decrease of the fluorine concentration. The hydrogen concentration decreases a lot at the beginning of the depth profile and remains almost constant throughout the remaining depth profiles. The carbon concentration increases gradually until reaching steady state conditions after the sputtering of the PTFE layers. The results for sample #4 with the thick PE and PTFE layers are similar. Only the trend for the fluorine intensity is slightly different, with a quasi-plateau corresponding to the two first PTFE layers and a further increase for the third and thickest PTFE layer. Using 20 keV Ar^+^ ions for sputtering improves the depth profile slightly, but as expected from Figure 5 the different layers cannot be properly distinguished. The two first PTFE layers form a first peak which is only slightly separated from a second peak corresponding to the third PTFE layer. Also, the peak maxima are less than a factor two above the fluorine contribution from atomic mixing. Results for the other polymer samples are similar. For comparison purposes, only the depth profile of sample #6 obtained for 20 keV Ar^+^ is shown. For PMMA, the oxygen signal has exactly the same trend than fluorine for the PTFE layers. Although carbon and hydrogen concentrations differ for the PE/PTE and PE/PMMA samples, their intensities do not differ significantly and the overall trend is identical. For all depth profiles the decay of fluorine or oxygen intensities after the sputtering of PTFE or PMMA layers is only slow.

Hence, for 20 keV Ne^+^ or Ar^+^ ion bombardment organic layers separated by 10 nm cannot be separated at all. For layers separated by 20 nm some distinction is possible and becomes apparent for a 40 nm distance between the layers (Figure 6b vs Figure 6c). For the latter, especially Ar^+^ ion bombardment leads to clearly visible separation of the layers compared to Ne^+^ irradiation, showing some significant advantage over Ne^+^. So, for imaging conditions in ion microscopy organic layers separated by 40 nm can be distinguished, but for 20 keV Ne^+^ it is really the minimum distance that is resolvable. Some separation is possible for 20 nm inter-layer distances, although the separation is not clear.

### Depth profiling with 1 keV Ne^+^ and Ar^+^

**Figure 7:**
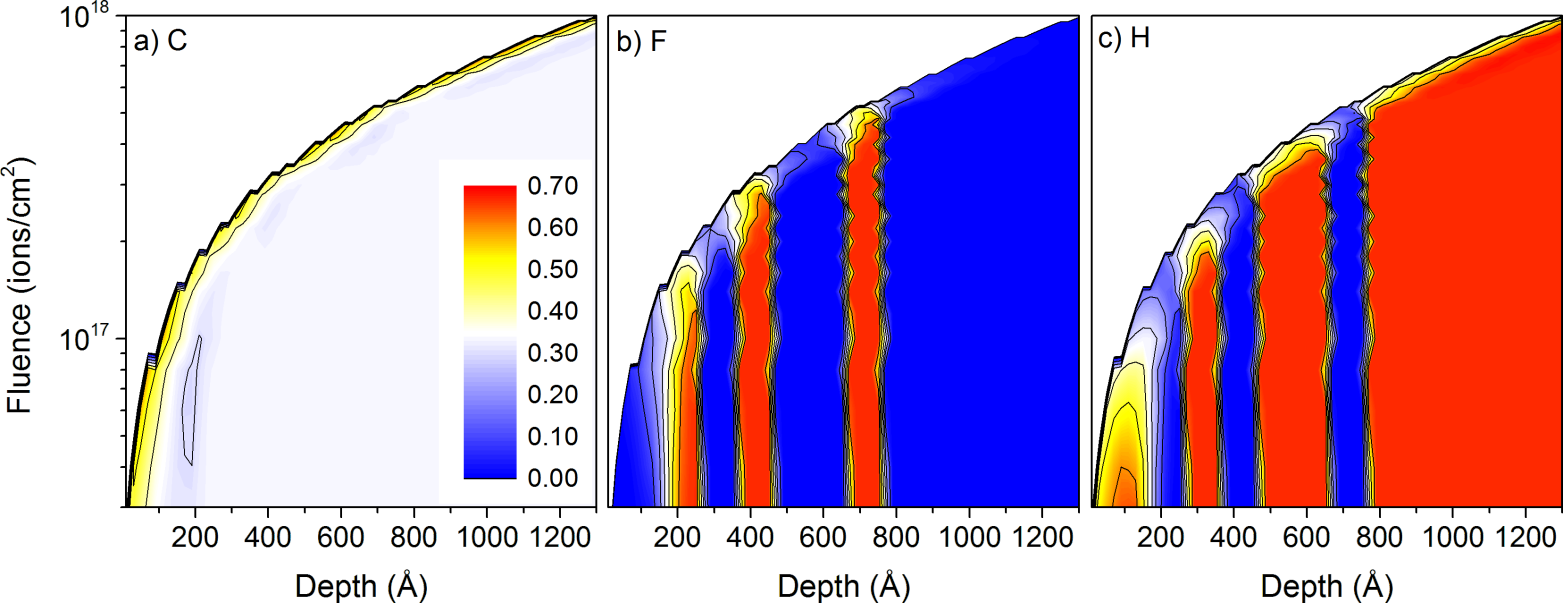
Sample composition as a function of depth and primary ion fluence for 1 keV Ne^+^ irradiation of sample #1 with the thin-layer configuration: a) for carbon, b) for fluorine, and c) for hydrogen. The colour scale shows concentrations from 0 to 70% and is identical for all species.

For 1 keV Ne^+^ and Ar^+^ beams, the lateral resolution will be reduced compared to the 20 keV conditions, but depth profiling capabilities are largely improved. For sample #1, the carbon concentration is increased close to the sample surface but it is not modified at larger depths like for the 20 keV irradiation (Figure 7). The mixing of fluorine and hydrogen between the PTFE and PE layers is also limited to a depth of 10 nm to 20 nm, helping a lot to keep the different layers separated. The fluorine surface concentration decreases from about 50% in the middle of the PTFE layer to about 8% in between the PTFE layers separated by 10 nm and to about 3% in between the PTFE layers separated by 20 nm (Figure 7b). Similarly the hydrogen surface concentration decreases significantly from 35% to 45% in the PE layers to 2% to 14% in the PTFE layers. The hydrogen concentration is highest in the first PTFE layer and lowest in the third one (Figure 7c). The results for 1 keV Ar^+^ bombardment are not shown, but the atomic mixing is further reduced. The fluorine surface concentration remains identical in the PTFE layers but decreases to 2% and 5% in between the layers. Hence, there is no huge difference in concentrations between the 20 nm and 40 nm inter-layer distances. The hydrogen surface concentration is about 45% in all PE layers and decreases to 0.3% and 5% in the PTFE layers. Here the change between Ne^+^ and Ar^+^ is larger than for fluorine.

**Figure 8:**
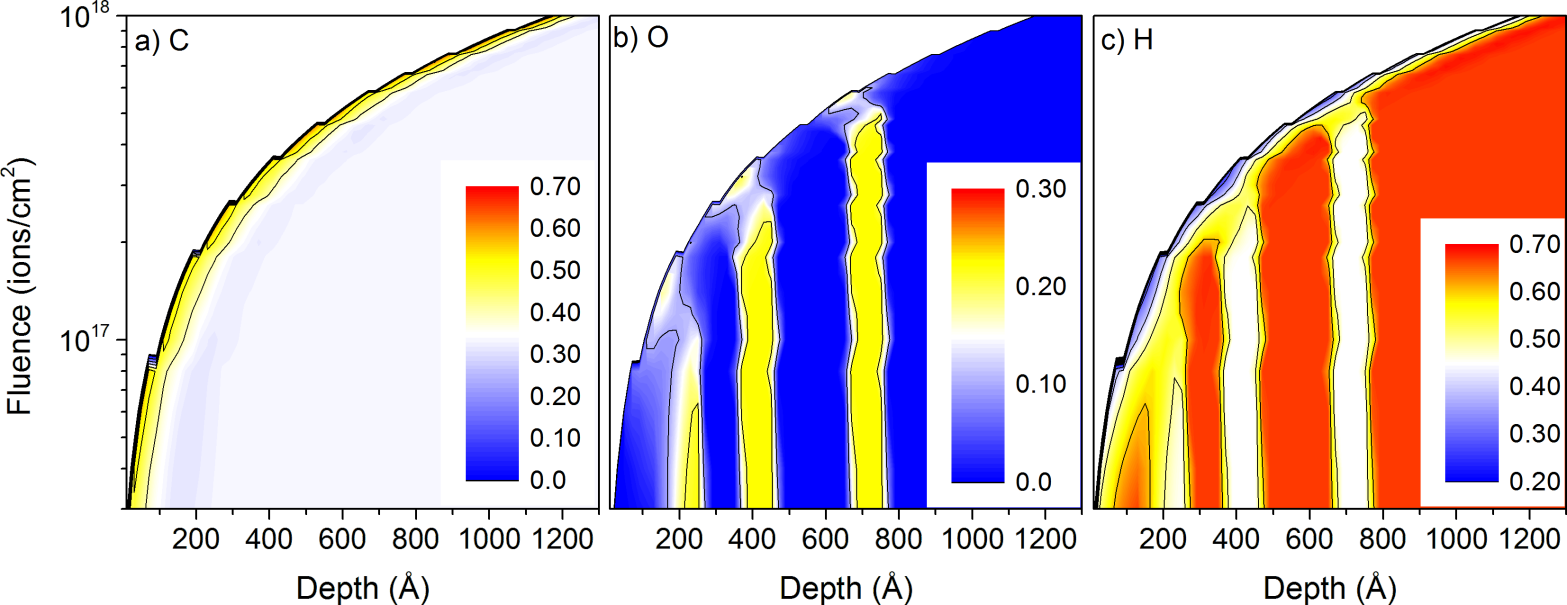
Sample composition as a function of depth and primary ion fluence for 1 keV Ne^+^ irradiation of sample #3 with the thin-layer configuration: a) for carbon, b) for oxygen, and c) for hydrogen. The colour scales are different for the three species.

For the PMMA sample #3, the carbon surface concentration is identical to the one of PTFE sample #1 and has a constant value of about 57% throughout the whole depth profile (Figure 8). The oxygen surface concentration in the PMMA layers is reduced to about 18% for all PMMA layers, compared to the bulk concentration of 22%. 10 nm below the surface, the oxygen concentration is even lower with about 12%. This behaviour was not obtained for fluorine in PTFE. In between the PMMA layers, the oxygen concentration reduces to 3% to 8%, the lower concentration being obtained for the larger inter-layer distance. The hydrogen surface concentration is changing less between the different layers with surface concentrations of about 40% in PE and about 28% PMMA. These values differ significantly from the bulk concentrations of 44% in PMMA and 67% in PE. For all polymers the sputtering with Ne^+^ leads to a material which is heavily enriched in carbon, although the enrichment is not as pronounced than for He^+^ irradiation where surface concentrations of about 67% are observed.

For 1 keV Ar^+^ irradiation, the carbon surface concentration is similar to the Ne^+^ irradiation. The oxygen shows also a very similar behaviour with surface concentrations about 18% in the PMMA layers and 1% to 5% in between them. It is also decreased to about 12% at a distance of 5–7 nm below the surface in the PMMA layer. For Ne^+^ and Ar^+^, this is probably due to the primary ion range of 6 nm to 8 nm. For hydrogen there are some differences in surface concentration between the PMMA and PE layers, with about 33% for PMMA and about 40% in between. This is a significantly smaller change than for Ne^+^. Overall, the modifications in surface concentrations induced by Ne^+^ and Ar^+^ are much smaller than those induced by the He^+^ bombardment.

**Figure 9:**
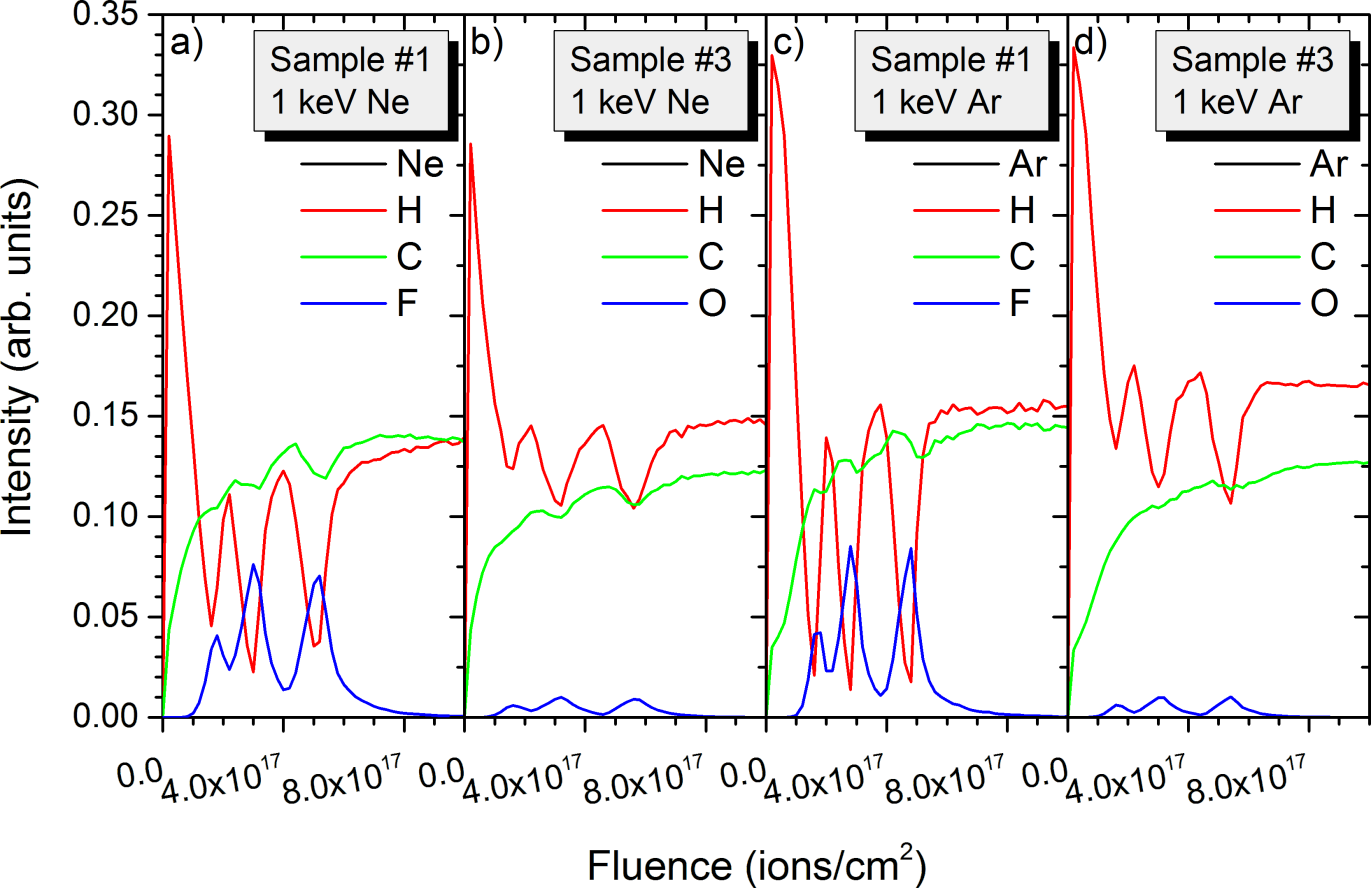
Simulated depth profiles for a) 1 keV Ne^+^ irradiation of sample #1, b) 1 keV Ne^+^ irradiation of sample #3, c) 1 keV Ar^+^ irradiation of sample #1, and d) 1 keV Ne^+^ irradiation of sample #3. All samples have inter-layer spacing of 10 nm and 20 nm.

Similar to the 20 keV irradiation, depth profiles have been extracted from the 2D data. Results are shown not only for the data presented in Figure 7 and Figure 8, but also for other conditions to get as broader overview of the influence of experimental conditions and sample composition on the sputtering processes. For the PTFE containing sample #1, the intensity changes in hydrogen and fluorine reveal well the multi-layered structure of the sample (Figure 9a). Both intensities decrease by a factor 4 to 7 in between the different layers. The hydrogen intensity shows also a decrease at the beginning of the sputtering process which is related to the preferential sputtering of hydrogen compared to carbon and fluorine. For the first PTFE layer, the fluorine and carbon intensities are still smaller than for the following ones. The layer is in the pre-equilibrium regime where preferential sputtering and atomic mixing have not attained their equilibrium values. Sputtering the same sample with Ar^+^ produces similar results (Figure 9c). The evolution of the intensities at the beginning of the sputtering process is similar to the Ne^+^ data. Only the higher mass combined with the lower implantation depth lead to a better separation of the different polymer layers. The ratios between intensity maxima and minima for the different layers increase to a factor 4–8 for fluorine and a factor 8–13 for hydrogen. Hence, the improvement is most pronounced for hydrogen. For both primary ion species, the carbon intensities show only minor variations between the different layers and cannot be used to differentiate between the PTFE and PE layers.

For the PMMA containing sample #3, the variations of the hydrogen intensity between PMMA and PE layers is less pronounced than for sample #1 (Figure 9b). This is expected since both PMMA and PE contain this element and PTFE not. However, the evolution of the different intensities at the beginning of the sputter process (e.g., the pre-equilibrium regime) is similar for both samples. The intensities of oxygen compared to fluorine are smaller due to the smaller oxygen concentration in PMMA compared to fluorine in PTFE. The ratios of maximum to minimum intensities of a factor 3–10 in the different layers are also comparable to the fluorine results. The variations in the hydrogen intensity are much smaller and are limited to a factor 1.5. This ratio is identical to the ratio of hydrogen concentrations in PE and PMMA (67% and 44%). The differences between Ar^+^ and Ne^+^ bombardment for sample #3 are identical to those of sample #1, the heavier species leading to a better separation of the different layers (Figure 9d).

**Figure 10:**
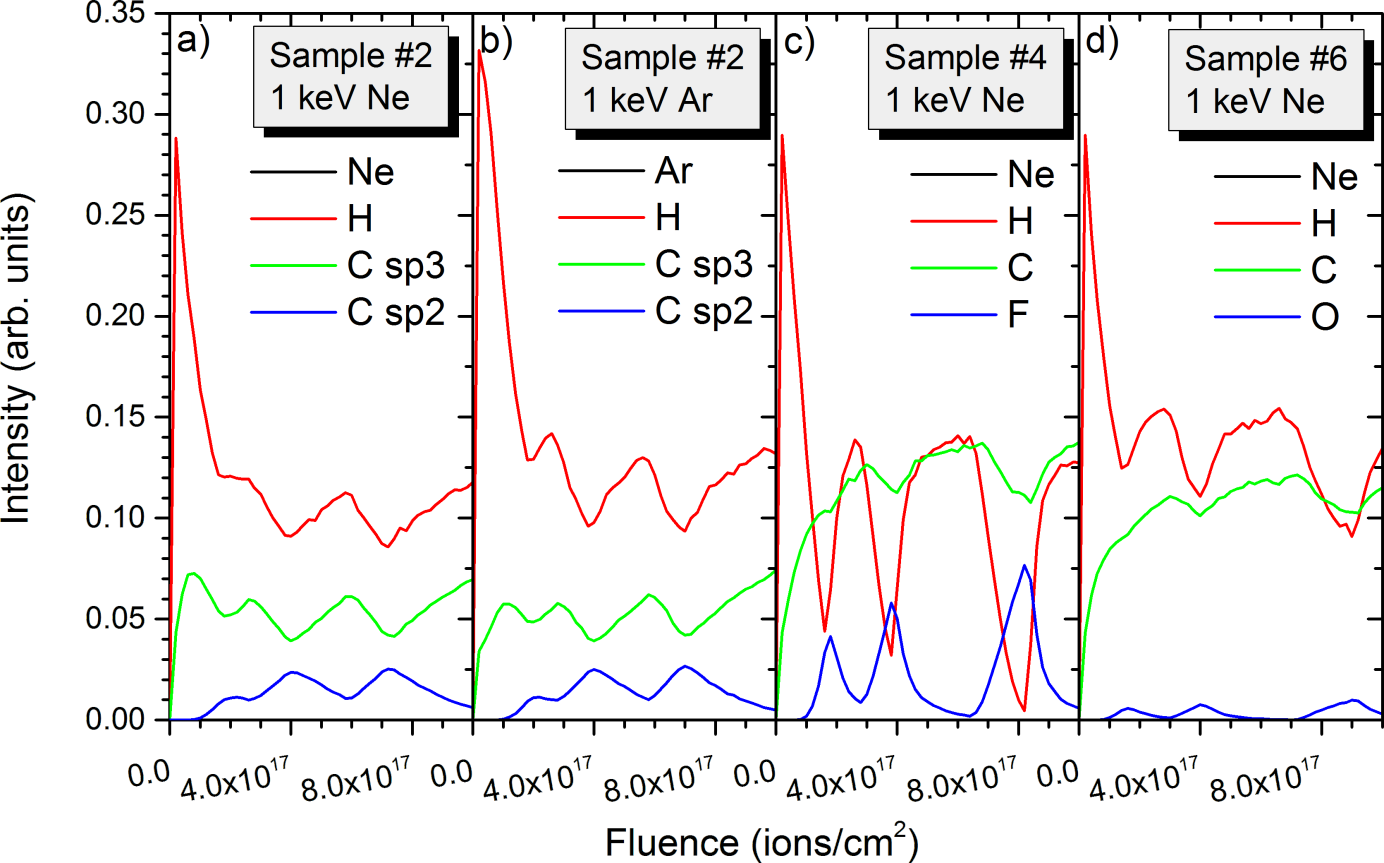
Simulated depth profiles for a) 1 keV Ne^+^ irradiation of sample #2, b) 1 keV Ar^+^ irradiation of sample #2, c) 1 keV Ne^+^ irradiation of sample #4, and d) 1 keV Ne^+^ irradiation of sample #6. Sample #2 has an inter-layer spacing of 10 nm and 20 nm, and samples #4 and #6 have an inter-layer spacing of 20 nm and 40 nm.

For samples #1 and #3, the fluences required to reach a given layer are more or less identical for both samples and both primary ion species. A small difference is observed between the two samples with a fluence of 5.2 × 10^17^ ions/cm^2^ required for the third PTFE layer in sample #1 and a fluence of 5.6 × 10^17^ ions/cm^2^ for the third PMMA layer in sample #3. Both examples are given for Ne^+^ bombardment. For sample #2, the fluence required to reach the third PS layer is significantly higher (7.4 × 10^17^ ions/cm^2^), showing that PS is more difficult to sputter than PTFE and PMMA (Figure 10a). The overall behaviour of the intensities in the pre-equilibrium regime is however identical. In experiments, differentiating the different layers by depth profiling using monatomic ion species is more difficult because sp3 and sp2 carbon atoms cannot be distinguished. One solution would be to use isotopic labelling with ^12^C and ^13^C for the different polymers.

For an increased distance between the polymer layers, the separation between the layers increases (Figure 10c and 10d). For the PTFE containing sample #4, the ratio of maximum to minimum intensities ranges between a factor 5 and 28, which is significantly more than for the smaller inter-layer distances of Figure 9a. Especially for the 40 nm inter-layer spacing the dynamic range is significantly improved. For the hydrogen intensity, the ratio is also increased for the larger inter-layer spacing, showing a factor 5 to 30 for the small and large distance. A similar evolution of the separation between the layers is observed for the PMMA containing sample #6. The ratio for the oxygen intensity is equal to 6 and 58 for the small and larger inter-layer spacing.

Hence, 2D or 3D depth profiling by 1 keV Ne^+^ ions allows distinguishing layers separated by 10 nm, the intensities at the beginning of the analysis up to a fluence always being influenced by the pre-equilibrium regime. In general results obtained by Ne^+^ bombardment are similar to Ar^+^ irradiation, the separation of the layers being slightly better for the Ar^+^ beam. The difference between the two is largest for the light elements where Ar^+^ outperforms Ne^+^ by up to a factor 8. Although producing better lateral resolutions, He^+^ bombardment is not suited for depth profiling applications. The very slow sputter yields combined with the important atomic mixing lead to a complete blurring out of the in-depth information. In general, the depth resolution is not as good as the 1 nm to few nm resolution obtained with below keV heavy primary ions [34] or cluster ion bombardment [35], but a good compromise between depth and lateral resolution. For few keV impact energies, depth resolution will be degraded but layers separated by 20 or 40 m can be still separated. If highest lateral resolutions are required and depth resolution is less of an issue, also higher impact energies can be chosen.

Results in this work have been obtained on selected multi-layered samples of different polymers. The results are not only valid for such polymer samples but can also be applied to other organic systems including biological samples, i.e., to systems which contain mainly relatively light chemical elements. For samples containing metal atoms, the atomic mixing could be changing. Examples include mixed organic–inorganic systems for organic solar cells or LEDs. A study on such systems is forthcoming.

### Numerical methods

Simulations on sputtering were carried out using the SD_TRIM_SP code [26] which is based on the simulation codes TRIM [36–37] and TRIDYN [38–39]. In addition to previous codes, SD_TRIM_SP includes the option to take the outgassing of atoms in a sample into account [40]. This is required for the simulation of helium, neon and argon ion bombardment of polymer samples. The diffusion coefficients for the different rare gas species have been taken from a previous work [30]. The irradiation was simulated for 1 keV and 20 keV ion impacts at normal incidence which corresponds to experimental conditions for depth profiling and imaging in SIMS. During the simulations, the KrC potential has been used for interatomic interactions, the Oen–Robinson model for electronic stopping and the Gauss–Mehler method with 16 pivots for integration. The surface binding energy is calculated using *sbe*(*i*,*j*) = 0.5(*Es*_i_ + *Es*_j_), where *sbe* is the surface binding energy for the target of consideration and *Es*_i_ is the atomic surface binding energy [26].

To study the influence of sample composition on sputtering and atomic mixing, six different multi-layered samples have been investigated. For all samples, polyethylene (PE) was used as bulk material. For each sample, six layers were put on top of the PE substrate, layers 1, 3 and 5 being always PE. Layers 2, 4, and 6 were made of polytetrafluoroethylene (PTFE), polystyrene (PS) or poly(methyl methacrylate) (PMMA). For samples 1 to 3, layers 1 and 5 have a thickness of 20 nm while the remaining layers have a thickness of 10 nm. For samples 4 and 6, layers 1, 3 and 6 are 20 nm thick, layers 2 and 4 are 10 nm thick and layer 5 is 40 nm thick. The composition and structure of the different samples is summarised in Table 1. The different samples have been chosen to get insights on the influence of polymer composition and structure, and layer thickness on sputtering and atomic mixing and the influence of experimental conditions on depth profiling capabilities.

## Conclusion

Ion microscopy on the helium ion microscope (HIM) provides higher lateral resolution than on classical SIMS instruments. At the same time the light rare gas primary ion species lead to higher implantation depth and different sputtering behaviour than primary ion species used in classical SIMS. In this work we investigated the depth profiling capabilities of He^+^ and Ne^+^ bombardment on multi-layered polymer samples. The influence of the primary ion species, of the polymer composition and of the structure and of the inter-layer distances on the depth profiles was investigated. On instruments, He^+^ beams provide the highest lateral resolution but their high implantation depth leading to important atomic mixing combined with the low sputter rates result in the total destruction of the initial sample structure. Hence, no information can be obtained from depth profiles. Ne^+^ ion beams behave similarly to Ar^+^ ions, with Ar^+^ producing some better depth resolution especially for light chemical elements. The latter serve as reference since they are used in classical SIMS experiments, and they have been investigated in more detail. For high impact energies at 20 keV typically used in secondary ion imaging conditions, Ne^+^ bombardment does not allow for a proper separation of the different layers. Polymer layers separated by a 10 nm or a 20 nm layer of another polymer cannot be separated at all. For inter-layer distances of 40 nm, two layers can be identified although no proper separation is obtained. At 1 keV Ne^+^ bombardment, corresponding to an impact energy typically used in depth profiling, the different polymer layers are well resolved. Depending on the inter-layer distances and the secondary ion intensity, the ratio between maximum and minimum intensity ranges from a factor 4 to 58. The factor 4 is obtained for a 10 nm distance between the layers, the factor 58 for a 40 nm spacing. Overall, 3D information at the 10 nm scale can be obtained for 1 keV Ne^+^ bombardment. For few keV impact energies, depth resolution will be degraded but layers separated by 20 or 40 nm can be still separated. If highest lateral resolutions are required and depth resolution is less of an issue, also higher impact energies can be chosen.
